# The effect of diagnosis-related group payment system on the quality of medical care for pelvic organ prolapse in Korean tertiary hospitals

**DOI:** 10.1371/journal.pone.0220895

**Published:** 2019-08-20

**Authors:** Myung Jae Jeon, Sung Pil Choo, Young Hwa Kwak, Dong Wook Kim, Eui Hyeok Kim

**Affiliations:** 1 Department of Obstetrics and Gynecology, Seoul National University College of Medicine, Seoul, South Korea; 2 Department of Obstetrics and Gynecology, Yonsei University College of Medicine, Seoul, South Korea; 3 Research and Analysis Team, National Health Insurance Service Ilsan Hospital, Goyang, South Korea; 4 Department of Obstetrics and Gynecology, National Health Insurance Service Ilsan Hospital, Goyang-si, South Korea; Medical University Graz, AUSTRIA

## Abstract

**Purpose:**

To assess changes in clinical practice patterns after implementing diagnosis-related group (DRG) payment system in July 2013 and its effect on the quality of care for pelvic organ prolapse (POP).

**Materials and methods:**

Using the 2011–2016 administrative database from National Health Insurance claim data, we reviewed medical information of 7362 patients who underwent hysterectomies for POP in Korean tertiary hospitals. We compared changes in several variables including length of stay, concomitant procedures, outpatient visits and readmission within 30 days after discharge, and retreatment for POP or stress urinary incontinence within postoperative 1 year before and after DRG system.

**Results:**

After the introduction of DRG system, the average length of stay decreased (7.74 ± 2.88 to 6.63 ± 2.18 days, p<0.001) without increasing readmission rates. However, the number of outpatient visits increased (2.78±2.33 to 2.98±2.47, p<0.001). Regarding concomitant procedures, the rates of colpopexy and midurethral slings significantly decreased (7.87% and 9.84% to 4.93% and 2.93%, respectively, all p<0.001). Even though there was no difference in the reoperation rates, pessary insertion for recurrent POP significantly increased after the introduction of DRG system (0.10% to 0.38%, p = 0.015).

**Conclusion:**

The implementation of DRG in Korean tertiary hospitals has led to increase of outpatient visits and reduced surgical management for POP, which indicates that the uniform application of DRG influences the quality of care for POP patients.

## 1. Introduction

Since its introduction in 1977, the National Health Insurance Program in Korea has paid health care providers on a fee-for-service basis. The fee-for-service reimbursement system has led to the rapid growth of health expenditures and changes in medical care such as the substitution of more profitable and less regulated services. To address these problems, the government adopted a pilot program utilizing diagnosis-related group (DRG) reimbursement for inpatient care in 1997. In 2002, this payment system was applied to seven disease groups (lens operation, tonsillectomy/adenoidectomy, appendectomy, inguinal/femoral hernia operation, hemorrhoidectomy, uterine/adnexa operation, cesarean delivery) through voluntary participation of health care institutions [1]. The DRG system became mandatory in hospitals and clinics on July 1, 2012, and was applied to general and tertiary hospitals on July 1, 2013.

Because DRG provides a fixed reimbursement for inpatient services, it encourages health care providers to reduce the length of stay or the intensity of services, which could influence the quality of care for patients. In spite of these concerns, several studies have reported that the Korean DRG system is effective in containing medical expenses with little negative impact on quality of care [1–3]. Further, it has also been reported that the implementation of the DRG-based payment system in the field of obstetrics and gynecology, specifically with regard to cesarean section, hysterectomy, and adnexectomy, in Korean tertiary hospitals led to reductions in the length of stay without increasing outpatient visits and readmission. Decreases in the rates of concomitant colpopexy and midurethral slings at the time of hysterectomy were also reported after introduction of the DRG [4].

Pelvic organ prolapse is the descent of one or more aspects of the vagina and uterus, including the anterior vaginal wall, posterior vaginal wall, uterus, or vaginal vault. Even though hysterectomy has historically been performed to treat uterine prolapse, hysterectomy alone is not adequate, and concomitant colpopexy should be performed to reduce the risk of recurrent POP [5–7]. Patients with POP are also at risk for postoperative stress urinary incontinence. POP and stress urinary incontinence coexist in up to 80% of patients [8], and stress urinary incontinence can occur after POP surgery even in patients without previous symptoms as a result of the correction of anatomical urethral kinking from advanced prolapse [9]. Therefore, a concomitant anti-incontinence surgery is often required to prevent postoperative stress urinary incontinence. Taken together, the decrease in the rates of concomitant procedures at the time of hysterectomy after introduction of the DRG may have an adverse effect on the quality of care for patients with POP.

In the current study, data were collected on the patients who underwent hysterectomy for POP with the extension of study period. Changes in clinical practice patterns after implementing the DRG-based payment system and its effect on the quality of care for POP were assessed.

## 2. Materials and methods

### 2.1. Database and data collection

The present study was a retrospective observational cohort study that included 43 tertiary hospitals in Korea that adopted the DRG-based payment system on July 1, 2013. Hospitals included in this study can be found in S1 Table.

National Health Insurance claim data collected from January 2011 to December 2016 were utilized. Patients included were those who were admitted to these hospitals from January 2011 to December 2015 to undergo hysterectomy for POP. Participants were identified by the Korea Classification of Diseases, 6th Edition diagnostic codes and Electronic Data Interchange procedure codes (Table 1). The study proposal was reviewed and approved by the institutional review board of National Health Insurance Service Ilsan Hospital in 2017 (NHIMC 2017-12-002-001).

**Table 1:** List of KCD-6 diagnostic codes corresponding to pelvic organ prolapse including uterine prolapse and EDI codes for hysterectomy and procedures to correct prolapse or stress urinary incontinence.

**KCD-6 Codes**	**Diagnosis**
N81.2	Incomplete uterovaginal prolapse
N81.3	Complete uterovaginal prolapse
N81.4	Uterovaginal prolapse, unspecified
N81.9	Female genital prolapse, unspecified
**EDI Codes**	**Hysterectomy Procedures**
R4145	Hysterectomy without lymphadenectomy (simple)
R4146	Hysterectomy without lymphadenectomy (complex)
R4202	Vaginal total hysterectomy of prolapsed uteri
R4203	Vaginal total hysterectomy with A-P repair of prolapsed uteri
**EDI Codes**	**Colporrhaphy Procedures**
Q3020	Correction of rectocele
R3620	Cystocele repair
R0408	Anterior colporrhaphy (with cystocele)
R0410	Posterior colporrhaphy (with rectocele)
R0412	A-P colporrhaphy (with cystocele and rectocele)
R4203	Vaginal total hysterectomy with A-P repair of prolapsed uteri
**EDI Codes**	**Colpopexy Procedures**
R4111	Colpopexy of abdominal approach (surgical)
R4112	Colpopexy of vaginal approach (surgical)
**EDI Codes**	**Midurethral Sling Procedure**
R3565	Operation for urinary incontinence (transvaginal approach)
**EDI Codes**	**Pessary Insertion Procedure**
R4113	Insertion of pessary

KCD, Korea Classification of Diseases; EDI, Electronic Data Interchange

To examine the effects of the DRG system on the quality of care for POP, several variables were evaluated including length of stay, concomitant procedures, subsequent outpatient visits, readmission, and retreatment for POP or stress urinary incontinence. Owing to limitations of the data, the only clinical characteristics of patients were age, economic status, and diagnosis of diabetes mellitus and/or hypertension. Length of stay was measured using admission and discharge dates. The numbers of outpatient visits and readmission rates within 30 days after discharge from the hospital were assessed. Rates of concomitant procedures and retreatment for POP or stress urinary incontinence within the first postoperative year were also examined. Electronic Data Interchange codes corresponding to colporrhaphy, colpopexy, pessary insertion, and midurethral slings were used for the analysis (Table 1).

### 2.2. Statistics analysis

The distribution of each categorical variable was examined using an analysis of frequencies and percentages, and chi-square tests were performed to examine the association between variables and the DRG payment system. Two-sample t-tests were performed to compare the average value and standard deviation for continuous variables. All statistical analyses were performed using SAS version 9.4 (SAS Institute, Inc.; Cary, NC, USA). A p-value of <0.05 was considered statistically significant.

## 3. Results

A total of 7362 patients were identified for inclusion in this study, with 3912 who underwent hysterectomy for POP before and 3450 who underwent hysterectomy for POP after the introduction of the DRG payment system. This represents a decrease of 11.8% in the total number of surgeries after the introduction of DRG. The patient group that underwent the operations after the introduction of the DRG system was significantly older but had fewer medical comorbidities such as diabetes and hypertension than the patient group undergoing surgery before the introduction of the DRG. There was no significant difference in the income level of the two groups (Table 2).

**Table 2:** Characteristics of the study population.

Variables	Before DRG (n = 3912)	After DRG (n = 3450)	p-value
Age (year)	65.69 ± 9.93	66.53 ± 9.45	<0.001*
Age (year)			<0.001*
≤ 40	43 (1.10)	16 (0.46)	
41–50	270 (6.90)	181 (5.25)	
51–60	686 (17.54)	560 (16.23)	
61–70	1339 (34.23)	1253 (35.32)	
71–80	1360 (34.76)	1238 (35.88)	
≥ 81	214 (5.47)	202 (5.86)	
Income level			0.217
Medical aid	228 (5.83)	155 (4.49)	
1–20%	492 (12.58)	456 (13.22)	
21–40%	410 (10.48)	363 (10.52)	
41–60%	505 (12.91)	456 (13.22)	
61–80%	346 (8.84)	308 (8.93)	
≥ 81%	1931 (49.36)	1712 (49.62)	
Diabetes mellitus	484 (12.37)	129 (3.74)	<0.001*
Hypertension	1102 (28.17)	870 (25.22	0.004*

Values are presented as mean ± standard deviation or n (%). DRG, diagnosis-related group.

*Statistically significant

After the introduction of the DRG system, the average length of stay significantly decreased (7.74 ± 2.88 to 6.63 ± 2.18 days, p<0.001). With regard to concomitant procedures, the rates of colpopexy and midurethral slings significantly decreased (8.13% and 10.71% to 5.04% and 3.77%, respectively, all p<0.001), while colporrhaphy rates slightly increased (Table 3). The decline in the rates of concomitant colpopexy and midurethral sling procedures did not change significantly in 2015, when a portion of operation fee for these procedures became reimbursable (the rates of colpopexy and midurethral sling in 2015 were 3.79% and 3.58%, respectively; all p<0.001 compared to those before the introduction of DRG system).

**Table 3:** Changes in the length of hospital stay and concomitant procedures after the introduction of diagnosis-related group payment system.

Variables	Before DRG (n = 3912)	After DRG (n = 3450)	p-value
Length of hospital stay (days)	7.74 ± 2.88	6.63 ± 2.18	<0.001*
Concomitant procedures			
Colporrhaphy	3575 (91.39)	3180 (92.17)	<0.001*
Colpopexy	318 (8.13)	174 (5.04)	<0.001*
Midurethral slings	419 (10.71)	130 (3.77)	<0.001*

Values are presented as mean ± standard deviation or n (%). DRG, diagnosis-related group.

*Statistically significant

The number of postsurgical outpatient visits significantly increased after the introduction of the DRG system (2.78±2.33 to 2.98±2.47, p<0.001) though readmission rates did not change. There was no difference in the reoperation rates for POP and stress urinary incontinence before and after introduction of the DRG. However, pessary insertion rates significantly increased after introduction of the DRG (0.10% to 0.38%, p = 0.015) (Table 4).

**Table 4:** Changes in outpatient visits, readmission, and retreatment for pelvic organ prolapse or stress urinary incontinence after the introduction of diagnosis-related group payment system.

Variables	Before DRG (n = 3912)	After DRG (n = 3450)	p-value
Outpatient visits within 30 days after discharge	2.78 ± 2.33	2.98 ± 2.47	<0.001*
Readmission within 30 days after discharge	255 (6.52)	210 (6.09)	0.288
Retreatment for POP or SUI within 1 year	65 (1.66)	65 (1.85)	0.530
Retreatment for POP	31 (0.79)	36 (1.01)	0.310
Reoperation	27 (0.69)	23 (0.63)	1.000
Pessary insertion	4 (0.10)	13 (0.38)	0.015*
Reoperation for SUI	34 (0.87)	29 (0.84)	0.995

Values are presented as mean ± standard deviation or n (%). DRG, diagnosis-related group; POP, pelvic organ prolapse; SUI, stress urinary incontinence.

* Statistically significant

## 4. Discussion

In this retrospective cohort study, changes in clinical practice patterns after implementation of a DRG-based payment system for hysterectomy and the effect of the DRG on the quality of care for POP among Korean tertiary hospitals were evaluated. Results indicate that the introduction of the DRG system reduced the length of stay, but resulted in an increase of postsurgical outpatient visits. In addition, rates of concomitant colpopexy and anti-incontinence procedures decreased after the introduction of the DRG, which led to the increase of retreatment rates using pessary for POP. These findings indicate that the uniform application of DRG for hysterectomy influences the quality of care for POP patients.

Contrary to the present study, a previous study found no adverse perioperative outcomes after the introduction of DRG for all hysterectomies for benign conditions: no increase in outpatient visits and readmission due to early discharge was noted [4]. This discrepancy may be due to the diversity of indications for hysterectomy. Although POP is one of the major indications for hysterectomy, it corresponds to just 11–13% of all hysterectomies, while the majority of hysterectomies are performed to treat other diseases including leiomyoma, adenomyosis, abnormal uterine bleeding, and benign ovarian neoplasm [4, 10]. While hysterectomies for other benign indications are usually performed for young women, POP surgeries are most often performed for women 70 years and older [11]. Older patients may have more underlying comorbidities and risks for perioperative complications, and may therefore require increased medical services [12–16]. One possible reason for the increase of outpatient visits in POP patients after the introduction of DRG system is that health care providers increased the use of outpatient care instead of increasing the length of hospital stay to maximize their profits while minimizing the reduction in the quality of care. The occurrence of perioperative complications not requiring hospitalization may also contribute to the increase of outpatient visits in these patients.

Another important finding is the decrease in the rates of concomitant colpopexy and midurethral slings after the introduction of DRG system. The Korean DRG payment covers all medical expenses for inpatient care except for meals, MRI, sonogram, extra charge for qualified specialist physicians and extra charge for rooms shared by less than six persons [1]. The medical costs for concomitant procedures could not be reimbursed until December 2014. Nonetheless, a prior study found that the rates of concomitant myomectomy and adnexectomy during cesarean section and colporrhaphy during hysterectomy did not change after the introduction of DRG system. However, there was a significant decrease in the rates of colpopexy and midurethral sling procedures at the time of hysterectomy [4]. The present study that only included the patients who underwent hysterectomy for POP during the extended period showed a similar result. The discrepancy according to the type of concomitant procedure may result from the difference in the cost of materials and level of difficulty. Myomectomy, adnexectomy, and colporrhaphy can be relatively easily conducted using only inexpensive suture materials. However, midurethral sling procedures require the use of an expensive surgical mesh, which cannot be reimbursed under the DRG system. Colpopexy can be performed with or without mesh, however, it requires a specialized surgical skill and is rarely performed by surgeons except for urogynecological subspecialists [17, 18]. Although there was a compensable increase in the rates of colporrhaphy after the introduction of DRG system, correction of anterior or posterior prolapse does not repair apical descent. Moreover, given the significant contribution of the vaginal apex to anterior and posterior vaginal support [19, 20], the best surgical correction of the anterior and posterior walls may fail unless the apex is adequately supported. One large population-based, retrospective cohort study has demonstrated that concomitant apical suspension procedure at the time of anterior or posterior colporrhaphy reduces the reoperation rates for recurrent prolapse at 10 years by half [7].

Nonetheless, our study showed no increase in the retreatment rates for POP and stress urinary incontinence except for pessary insertion after the introduction of DRG system. This result can be interpreted as follows. Recurrence rates of POP have been reported between 6% and 30% [21], and most of the recurrence is detected within the first postoperative year [22, 23]. However, many patients choose to monitor the prolapse or use a pessary instead of undergoing reoperation [24, 25]. The occurrence of stress urinary incontinence after POP surgery is also common, however, patients do not choose reoperation for this condition unless they have bothersome symptoms [26–28]. Instead, they can seek non-surgical treatment options such as lifestyle modification, pelvic floor muscle exercise and electrical stimulation.

## 5. Limitations and strengths

Our study has several limitations. First, the claim data of only 43 tertiary hospitals were examined, which does not represent the whole nation. In general, patients who are older and have more medical comorbidities or a more severe degree of POP are referred to tertiary hospitals. These patients’ characteristics may affect the results. Second, because the current study included the data on the patients who underwent hysterectomy, all changes in the clinical practice for POP since the introduction of the DRG system could not be assessed. The present study indicates that under the DRG system, health care providers may be reluctant to perform the operations for patients who require greater effort in medical care, as the patients undergoing operations after the introduction of DRG system had fewer medical comorbidities. Otherwise, the physicians may change their practice patterns for POP to operations that are not required to use the DRG payment system such as robot-assisted operations, which may result in an increase in health care expenditures. Third, the reoperation and pessary insertion rates as the retreatment outcomes for POP and stress urinary incontinence were assessed. Unfortunately, information on various non-surgical treatment options other than pessary insertion was not available using the National Health Insurance claim data. In addition, the follow-up duration was one year, which may not be long enough to evaluate reoperation rates considering the patients’ preference for non-surgical treatment options over reoperation.

Despite several limitations, the current study has many strengths. First, National Health Insurance claim data that included a large sample of patients and hospitals were used. Second, as of the time of writing, this is the first study to evaluate the effect of the introduction of DRG system on the quality of care for POP. Third, our results highlight the problems caused by the uniform application of the DRG system. To enhance the efficacy of DRG-based payments, it is essential to determine sufficiently homogenous groups of patients based on treatment cost. In contrast with hysterectomies for other benign indications, most of POP surgeries are performed for older women who incur significantly higher medical costs by requiring greater medical attention and having more comorbid conditions. In addition, hysterectomy is only one of the procedures used to treat POP, with additional procedures recommended for complete correction of POP. Therefore, the current Korean DRG system for hysterectomy needs to be reformed to provide sufficient medical care to the POP patients.

## 6. Conclusions

The implementation of DRG for hysterectomy in Korean tertiary hospitals has led to increase of outpatient visits and reduced surgical management of POP. Specifically, the rates of concomitant colpopexy and midurethral slings requiring a specialized skill or the use of an expensive material significantly decreased. Although these changes did not increase the rates of reoperation for recurrence POP and stress urinary incontinence within the first postoperative year, a further study will be required to evaluate the long-term outcomes.

## Supporting information

S1 Table The name of tertiary hospitals in Korea. (DOCX)

## References

[1] Kwon S. Payment system reform for health care providers in korea. Health policy and planning. 2003; 18: 84–92. 10.1093/heapol/18.1.84 12582111

[2] Kim JW, Shin DW, Chae JJ, Kim JY, Park SG. Impact of the new payment system on laparoscopic appendectomy in korea. The Journal of surgical research. 2015; 199: 338–44. 10.1016/j.jss.2015.04.070 26025628

[3] Kwak SH, Kim DH, Kim JM, Kim JH, Kim WS, Kim S, et al Impact of the korean diagnosis-related groups payment system on the outcomes of adenotonsillectomy: A single center experience. Auris Nasus Larynx. 2018; 45: 504–7. 10.1016/j.anl.2017.07.005 28756097

[4] Jung YW, Pak H, Lee I, Kim EH. The effect of diagnosis-related group payment system on quality of care in the field of obstetrics and gynecology among korean tertiary hospitals. Yonsei medical journal. 2018; 59: 539–45. 10.3349/ymj.2018.59.4.539 29749137 PMC5949296

[5] Barbalat Y, Tunuguntla H. Surgery for pelvic organ prolapse: A historical perspective. Curr Urol Rep. 2012; 13: 256–61. 10.1007/s11934-012-0249-x 22528116

[6] Downing KT. Uterine prolapse: From antiquity to today. Obstetrics and gynecology international. 2012; 2012: 649459–9. 10.1155/2012/649459 22262975 PMC3236436

[7] Eilber K, Alperin M, Khan A, Wu N, Pashos C, Clemens J, et al Outcomes of vaginal prolapse surgery among female medicare beneficiaries: The role of apical support. Obstetrics & Gynecology. 2013; 122: 981–7. 10.1097/AOG.0b013e3182a8a5e4 PMC3857933 24104778

[8] Bai SW, Jeon MJ, Kim JY, Chung KA, Kim SK, Park KH. Relationship between stress urinary incontinence and pelvic organ prolapse. Int Urogynecol J. 2002; 13: 256–60. 10.1007/s001920200053 12189431

[9] Brubaker L, Cundiff GW, Fine P, Nygaard I, Richter HE, Visco AG, et al Abdominal sacrocolpopexy with burch colposuspension to reduce urinary stress incontinence. The New England Journal of Medicine. 2006; 354: 1557–66. 10.1056/NEJMoa054208 16611949

[10] Wu JM, Wechter ME, Geller EJ, Nguyen TV, Visco AG. Hysterectomy rates in the United States, 2003. Obstet Gynecol 2007; 110: 1091–5. 10.1097/01.AOG.0000285997.38553.4b 17978124

[11] Yuk J, Lee JH, Hur J, Shin J. The prevalence and treatment pattern of clinically diagnosed pelvic organ prolapse: A korean national health insurance database-based cross-sectional study 2009–2015. Scientific reports. 2018; 8: 1334–6. 10.1038/s41598-018-19692-5 29358718 PMC5778022

[12] Oh S, Shin SH, Kim JY, Lee M, Jeon MJ. Perioperative and postoperative morbidity after sacrocolpopexy according to age in korean women. Obstetrics & Gynecology Science. 2015; 58: 59–64. 10.5468/ogs.2015.58.1.59 PMC4303754 25629020

[13] Richter HE, Goode PS, Kenton K, Brown MB, Burgio KL, Kreder K, et al The effect of age on Short‐Term outcomes after abdominal surgery for pelvic organ prolapse. Journal of the American Geriatrics Society. 2007; 55: 857–63. 10.1111/j.1532-5415.2007.01178.x 17537085

[14] Lee JFY, Leow CK, Lau WY. Appendicitis in the elderly. ANZ Journal of Surgery. 2000; 70: 593–6. 10.1046/j.1440-1622.2000.01905.x 10945554

[15] Kim K, Lee SC, Lee SK, Choi B, Jeong W, Kim S. Does korea's current diagnosis-related group-based reimbursement system appropriately classify appendectomy patients? Annals of surgical treatment and research. 2016; 91: 66–73. 10.4174/astr.2016.91.2.66 27478811 PMC4961888

[16] Mason A, Or Z, Renaud T, Street A, Thuilliez J, Ward P. How well do diagnosis-related groups for appendectomy explain variations in resource use? an analysis of patient-level data from 10 european countries. Health Economics. 2012; 21: 30–40. 10.1002/hec.2836 22815110

[17] Kantartzis K, Turner L, Shepherd J, Wang L, Winger D, Lowder J. Apical support at the time of hysterectomy for uterovaginal prolapse. Int Urogynecol J. 2015; 26: 207–12. 10.1007/s00192-014-2474-y 25182150

[18] Fairchild Pamela S., MD|Kamdar Neil S., MA|Berger Mitchell B., MD, PhD|Morgan Daniel M., MD. Rates of colpopexy and colporrhaphy at the time of hysterectomy for prolapse. American Journal of Obstetrics and Gynecology. 2016; 214: 262.e7. 26366666 10.1016/j.ajog.2015.08.053 PMC4744488

[19] Rooney K, Kenton K, Mueller ER, FitzGerald MP, Brubaker L. Advanced anterior vaginal wall prolapse is highly correlated with apical prolapse. Am J Obstet Gynecol. 2006; 195: 1837–40. 10.1016/j.ajog.2006.06.065 17132485

[20] Lowder JL, Park AJ, Ellison R, Ghetti C, Moalli P, Zyczynski H, et al The role of apical vaginal support in the appearance of anterior and posterior vaginal prolapse. Obstet Gynecol. 2008; 111: 152–7. 10.1097/01.AOG.0000297309.25091.a0 18165404

[21] Dällenbach P. To mesh or not to mesh: A review of pelvic organ reconstructive surgery. International journal of women's health. 2015; 7: 331–43. 10.2147/IJWH.S71236 25848324 PMC4386830

[22] Weber AM, Walters MD, Piedmonte MR, Ballard LA. Anterior colporrhaphy: A randomized trial of three surgical techniques. American Journal of Obstetrics and Gynecology. 2001; 185: 1299–306. 10.1067/mob.2001.119081 11744900

[23] Dietz H, Hankins K, Wong V. The natural history of cystocele recurrence. Int Urogynecol J. 2014; 25: 1053–7. 10.1007/s00192-014-2339-4 24556972

[24] Barber MD, Brubaker L, Burgio KL, Richter HE, Nygaard I, Weidner AC, et al Comparison of 2 transvaginal surgical approaches and perioperative behavioral therapy for apical vaginal prolapse: The OPTIMAL randomized trial. JAMA. 2014; 311: 1023–34. 10.1001/jama.2014.1719 24618964 PMC4083455

[25] Suh DH, Jeon MJ. Risk factors for the failure of iliococcygeus suspension for uterine prolapse. European Journal of Obstetrics and Gynecology. 2018; 225: 210–3. 10.1016/j.ejogrb.2018.05.001 29747142

[26] Brubaker L, Nygaard I, Richter HE, Visco A, Weber AM, Cundiff GW, et al Two-year outcomes after sacrocolpopexy with and without burch to prevent stress urinary incontinence. Obstetrics and gynecology. 2008; 112: 49–55. 10.1097/AOG.0b013e3181778d2a 18591307 PMC2614233

[27] Wei JT, Nygaard I, Richter HE, Nager CW, Barber MD, Kenton K, et al A midurethral sling to reduce incontinence after vaginal prolapse repair. The New England Journal of Medicine. 2012; 366: 2358–67. 10.1056/NEJMoa1111967 22716974 PMC3433843

[28] Jeon M, Kim J, Moon Y, Bai S, Yoo E. Two-year urinary outcomes of sacrocolpopexy with or without transobturator tape: Results of a prolapse-reduction stress test-based approach. Int Urogynecol J. 2014; 25: 1517–22. 10.1007/s00192-014-2410-1 24819329

