# Supplementary material for: The effect of diagnosis-related group payment system on the quality of medical care for pelvic organ prolapse in Korean tertiary hospitals
Source: PLoS One. 2019 Aug 20;14(8):e0220895. doi: 10.1371/journal.pone.0220895 (PMC6701833; doi:10.1371/journal.pone.0220895)
Supplement: S1 Table — (DOCX) [file pone.0220895.s001.docx]

Supplementary 1. The name of tertiary hospitals in Korea

| Area of medical care | Tertiary hospitals |
| --- | --- |
| Capital area | The Catholic university of Korea Seoul St. Mary's Hospital  The Catholic university of Korea Yeouido St. Mary's Hospital  Konkuk University Medical Center  Kyung Hee University Medical Center  Korea University Guro Hospital  Korea University Anam Hospital  Samsung Medical Center/ Kangbuk Samsung Hospital  Seoul National University Hospital/ Asan Medical Center  Soon Chun Hyang University Hospital  Severance Hospital/ Gangnam Severance Hospital  Ewha Womans University Medical Center  Inje University Sanggye Paik Hospital  Chung-ang University Hospital  Hanyang University Medical Center |
| Western region of Gyeongi province | Soon Chun Hyang University Hospital Bucheon  Gacheon University Gil Medical Center  Inha University Hospital  Hallym University Medical Center |
| Southern region of Gyeongi | Korea University Ansan hospital  Seoul National University Bundang Hospital  Ajou University Hospital |
| Gangwon province | Wonju Severance Christian Hospital |
| Chungcheong province | Chungbuk National University Hospital  Dankook University Hospital  Soon Chun Hyang University Hospital Cheonan  Chungnam National University Hospital |
| Jeolla province | Chonnam National University Hospital  Chosun University Hospital  Chonnam National University Hwasun Hospital  Wonkwang University Hospital  Chonbuk National University Hospital |
| Gyeongsang province | Kyungpook National University Hospital  Keimyung University Dongsan Medical Center  Daegu Catholic University Medical Center  Yeungnam University Medical Center  Gyeongsang National University Hospital  Kosin University Gospel Hospital  Dong-A University Hospital  Pusan National University Hospital  Inje University Busan Paik Hospital |
